# β-Caryophyllene Ameliorates MSU-Induced Gouty Arthritis and Inflammation Through Inhibiting NLRP3 and NF-κB Signal Pathway: In Silico and In Vivo

**DOI:** 10.3389/fphar.2021.651305

**Published:** 2021-04-23

**Authors:** Wan-Yang Li, Fan Yang, Ji-Hua Chen, Guo-Feng Ren

**Affiliations:** ^1^ Xiangya School of Public Health, Central South University, Changsha, China; ^2^ School of Chinese Traditional Medicine, Shandong University of Traditional Chinese Medicine, Jinan, China

**Keywords:** β-caryophyllene, gout, monosodium urate, nucleotide-binding oligomerization domain-like receptor protein 3, nuclear factor kappa-B

## Abstract

Gouty arthritis serves as an acute reaction initiated by the deposition of monosodium urate (MSU) crystals around the joints. In this study, the anti-inflammatory effects of phytochemical β-caryophyllene on MSU crystal-induced acute gouty arthritis *in vivo* and in silico were explored. Through bioinformatics methods and molecular docking, it screened the specific influence pathway of β-caryophyllene on gout. Certain methods including enzyme-linked immunosorbent assay, western blotting, and immunohistochemical staining were adopted to quantify. β-caryophyllene significantly reduced inflammation and function of ankle joints in MSU Crystals-induced gouty arthritis rats, while decreasing serum cytokine levels. Furthermore, it inhibited the expressions of NLRP3, Caspase-1, ASC, TLR4, MyD88, p65, and IL-1β in the synovial tissue so as to reduce inflammation and protect ankle joints’ function. A new research approach in which β-caryophyllene treatment to acute attacks of gout is provided through the research results.

## Introduction

Gout is regarded as an inflammatory reaction initiated by the deposition of monosodium urate (MSU) crystals around the joints. It affects about 0.1–10% of adults. Moreover, the prevalence rate of men is higher than women (Jhang et al., 2018). As for the development of gout, the inflammatory response ranks first. The intrinsic mechanism should be summarized as that MSU crystals induce intra-articular inflammation through activating the complement system and recruiting neutrophils and macrophages. Consequently, it gives rise to cartilage and synovial tissue damage, and it may eventually develop into joint damage and deformity (Kuo et al., 2015).

MSU crystals recruit nucleotide-binding oligomerization domain-like receptor protein 3 (NLRP3) to the release of inflammatory mediators, such as interleukin-1β (IL-1β), interleukin 6 (IL-6) and Tumor Necrosis Factor α (TNF) (Wang et al., 2019). Importantly, the massive release of IL-1β is deemed as an early feature of gout (Martinon, 2010; Mitroulis et al., 2013). Besides, oxidative stress is also involved in the inflammatory process. Based on the results drawn by previous studies, redox signaling molecules such as reactive oxygen species (ROS) is able to mediate the formation of NLRP3 inflammasome, thus interfering with NLRP3-mediated inflammatory responses (Martinon, 2010; Mitroulis et al., 2013). The NLRP3 inflammasome turns out to be significant in various inflammatory diseases. Moreover, it is observed that its abnormal activation is involved in inflammatory disease’s pathogenesis, such as Alzheimer’s disease, multiple sclerosis, obesity, diabetic complication, inflammatory bowel diseases and gout (Lim et al., 2020; Zhang et al., 2020). Toll-like receptors (TLRs) are defined as a significant class of recognition receptors in the innate immune system. Furthermore, it has been involved in the recognition of pathogen-associated molecular patterns to a large extent. Based on the recent evidence, it is suggested that TLRs might participate in MSU crystal’s identification and activation (Crisan et al., 2016; Liu et al., 2019). The TLRs are combined with Myeloid differentiation factor88 (MyD88), thus forming a complex, recruiting and causing inflammation reaction. All these have been realized through activating the transformation of growth factor-β (TGF-β) kinase activity, which activates the transcription factor nuclear factor kappa-B (NF-κB) and initiates the IL-1β-specific precursor pro-IL-1β gene transcription and expression. Based on the recent reports, it is suggested that gouty arthritis main clinical treatments are non-steroidal anti-inflammatory drugs (NSAIDs) and colchicine. However, the side effects set limitations on its clinical application (Qaseem et al., 2017). To be more specific, indomethacin may result in kidney toxicity in elderly patients. Moreover, the long-term use of colchicine is likely to inhibit bone marrow and hematopoietic function (Martinon, 2010).

From this perspective, numerous studies have highlighted that natural compounds should serve as the complementary support when treating inflammation-related disease (Aggarwal et al., 2013; Koh et al., 2018), especially gout (Jhang et al., 2018; Ruiz-Miyazawa et al., 2018). As a natural bicyclic sesquiterpene (Chen et al., 2011), β-caryophyllene is mainly found in natural plants such as cloves, cinnamon, and lemon. It is supposed to embrace biological activities, such as anti-inflammatory effects (El-Sheikh et al., 2019), anti-tumor (Ambroz et al., 2019), anti-oxidation (Al-Taee et al., 2019), and other pharmacological effects, which all turn out to be beneficial. Recently, the anti-rheumatoid of β-caryophyllene which can reduce systemic inflammation and oxidative stress levels has been reported in arthritic rats (Ames-Sibin et al., 2018). Despite of this, its effect on gouty arthritis remains unclear.

Molecular docking has been widely employed in leading identification to simulate the interaction between small-molecule ligands and receptor biomacromolecules. During this process, the “lock-key” principle of ligand-receptor interaction and the principle of “induction fit” by computer pattern recognition and optimization techniques are adopted respectively (Lohning et al., 2017). According to a large number of studies, computer-aided drug screening has managed to seek the best molecule combination, which has achieved success in active botanical components virtual screening and drug targets discorvery (Wang et al., 2017; Gong et al., 2018; Zihad et al., 2018; Saikia and Bordoloi, 2019). In this case, with the development of bioinformatics technology, it has become possible to extract the differentially expressed genes for specific diseases from open databases when conducting comprehensive research (Liang et al., 2016). The effect of β-caryophyllene on gouty arthritis has not been previously resolved, however, all studies concerning this topic have been reported merely *in Vitro* or *in Vivo*. As for the Silico research, it conforms to the results *in Vivo* experiments, suggesting that the phytochemical has exhibited powerful binding interaction with target proteins.

Therefore, based on the GeneCards database, this study screened the differential genes between gout and inflammation. Moreover, molecular docking was used for virtual screening so as to theoretically infer the stable binding targets of β-caryophyllene. Later, the model of acute gouty arthritis in rats was established to study the effects of β-caryophyllene on acute gouty arthritis treatment. Furthermore, it also explored its effects of anti-inflammatory signaling pathways with the aid of molecular biology research methods.

## Materials and Methods

### Animals

Male Sprague-Dawley rats (200 ± 20 g) were purchased from Pengyue, Jinan, China. All animals were maintained in a pathogen‐free environment under a light/dark cycle with a temperature of 22 ± 1°C and a humidity of 50 ± 10% for 12 h, allowing free access to diet and water. The experimental method was approved by the Animal Care Ethics and Use Committee of Xiangya School of Public Health Central South University (No.XYGW-2019-080) and performed according to the guidelines of the committee guidelines. Animals used in this study were maintained in accordance with the Guide for Care and Use of Laboratory Animals published by the National Institutes of Health guide (NIH Publications No. 8023, revised 1978) and the Policy of Animal Care and Use Committee of Central South University.

### The Animal Model of Acute Gouty Arthritis

The acute gouty arthritis model was induced by injection of MSU (≥98%, Sigma-Aldrich, United States) crystal (20 mg suspended in 1 ml sterile saline, injection 50 uL MSU suspension per rat) in the right ankle joint of rats (Zhou et al., 2018). Doses of β-Caryophyllene (≥98%, Sigma-Aldrich, United States, Supplementary Datasheet S1 for identification information) were selected to treat the model of arthritis rats which were divided into the following seven groups at random, with eight animals in each group: normal control group, MSU (model) group, MSU+β-Caryophyllene (BCP:100 mg/kg, once a day) group, MSU+β-Caryophyllene (BCP:200 mg/kg, once a day) group, and MSU+β-Caryophyllene (BCP:400 mg/kg, once a day) group, MSU + Indomethacin (Ind: 5 mg/kg, once a day) group, β-Caryophyllene (BCP:400 mg/kg, once a day) group. BCP (in MSU + BCP groups) or IND (as a positive control) was given to rats by gavage before the administration of MSU. The control group and model group received an equal volume of 0.9% saline (10 ml/kg). On day 7, the rats in the MSU experimental groups were injected with 50 μL MSU suspension into the right ankle joint cavity, while the control group were injected with an equal volume of normal saline in the right ankle cavity. After the MSU injection for 48 h, the rats in all the groups were killed by intraperitoneal injection with 10% chloral hydrate, and synovial tissue samples of all rats were divided into two sets. One set was flash‐frozen in liquid nitrogen and stored at −80°C. The remaining samples ankle joint were fixed in 4% paraformaldehyde buffer for paraffin embedding of synovial tissues.

### Assessment of Gouty Arthritis

The circumference of each rat’s right ankle was measured at the same position 4, 10, 24, and 48 h after modeling. Moreover, the degree of swelling was calculated. Degree of expansion = (model circumference after modeling - circumference of ankle joint before modeling)/circumference of ankle joint before modeling. Meanwhile, indicators of joint inflammation and dysfunction were evaluated by Coderre classification (Coderre and Wall, 1987): the ankle joint state and gait of the right leg were observed at 10, 24, and 48 h after the establishment of the model.

Concerning the grading criteria for the joint inflammation index, there should be Level 0: Normal; Level 1: Joint skin erythema, mild swelling, and visible bone marks; Level 2: The joints are red and swollen, and the bone marks disappear, but the swelling is limited to the joints. Level 3: Limbs are swollen outside the joint. The indicators of joint dysfunction index are as follows: Level 0: Normal; Level 1: difficult to land on the feet toes not open, and mild limp; Level 2: feet bent, toes backward, and obvious limp. Level 3: The foot completely leaves the ground, and three-legged gait.

The calibrated von Frey filament BIO-VF-M model from Bioseb (French Bioseb *in vivo* research instrument) was used for mechanical stimulation to evaluate the change of hind paw withdrawal threshold. The threshold is determined using the Dixon up and down method (Marcotti et al., 2018). The rat was placed in a transparent plastic cylinder on a metal mesh platform, and von Frey filaments were applied to the hind paw’s sole surface for 1 s. Rapid withdrawal of the hind paw during or immediately after the application is considered as a positive reaction. The threshold force required for the right hind paw was determined to cause withdrawal (50% withdrawal of the hind paw). Threshold data is expressed as the difference from the baseline value, and negative values indicate allodynia (Chaplan et al., 1994). The threshold was evaluated in rats before the right ankle joint injection of the MSU solution 6 and 12 h after the injection.

With the aid of the enzyme-linked immunosorbent assay (ELISA) kit (Wuhan Huamei Bioengineering Co., Ltd.), the rat serum was analyzed in a multi-functional enzyme labeler (Thermo Fisher Scientific) when measuring IL-1β, IL-6, and TNF content based on the protocol of kits. Synovial tissues were fixed in 4% paraformaldehyde after the decalcification and sectioned with hematoxylin and eosin (HE), thus carrying out further histological analysis.

### Assessment of Hepatic Toxicity Parameters

After the MSU injection for 48 h, each rat venous blood samples were collected for serum separation. Samples were stored at −20°C until further analysis. Alanine aminotransferase (ALT), aspartate aminotransferase (AST), and alkaline phosphatase (ALP) detection kits (Nanjing Jiancheng Bioengineering Institute, Nanjing, China) were used in strict accordance with the protocol of the manufacturer. Liver tissues were fixed in 4% paraformaldehyde, paraffin-embedded, and sectioned with hematoxylin and eosin (HE) for histological analysis.

### Bioinformatics Analysis

GeneCards (https://www.genecards.org/) is a comprehensive database of genes that summarizes gene information in detail, including disease-gene relationships, gene functions, gene mapping, and pathways for gene involvement. OMIM (Human Online Mendelian Inheritance, https://omim.org) is an authoritative database of human genes and genetic phenotypes, including the relationship between phenotypes and genotypes. From the aforementioned database, when using Perl 5.26.3 to extract, gene target information for gouty arthritis action will be obtained (see Supplementary Datasheet S2 for the data set involved in this research).

The String database (https://string-db.org/) is a database for searching interactions between proteins, including using experimental data and PubMed to collect information, integrating other databases and using bioinformatics methods to predict. In this study, a protein interaction network (PPI network) was established through STRING11.0 online analytical platform. In the meantime, R 3.5.2 Collect was used to calculate genetic information, thus obtaining protein interaction results.

As for GO functional enrichment analysis, it serves as a process of classifying the above-mentioned collected genes or proteins, and divides the gene or protein list into multiple parts. During this process, the classification criteria are defined according to genes function. Specifically, those genes who share similar functions are put together and associated with a biological phenotype. KEGG enrichment analysis is defined as a process of categorizing the genes, which are collected by integrating the gene pathway database (KEGG Pathway), and genes located in the same pathway, as well as summarizing and predicting the disease’s possible gene pathway mechanisms. This study adopts the R package “DOSE”, “clusterProfiler” and “pathview” for GO analysis, KEGG enrichment analysis, as well as Visual processing.

### Docking Studies

Molecular docking experiment was performed by the Discovery Studio 2016. Moreover, views of docking results were also completed through DS 2016. The docking analysis was carried out through CDOCKER Module. When dealing with docking studies, the X-ray crystallographic structures of NLRP3(PDB ID 6NPY), Caspase-1(PDB ID 2H4W), Nf-κB p65(PDB ID 1VKX), TLR4(PDB ID 2Z62), and MyD88(PDB ID4DOM) were taken as the protein structures. All these were obtained from the Protein Date Bank (https://www.rcsb.org/). To sum up, the structure of β-Caryophyllene (Compound CID 5281515) was downloaded from the PubMed website (https://pubchem.ncbi.nlm.nih.gov/). And the best docking poses were picked out after ranking and categorizing via CODOCKER Interaction Energy.

### Western Blot Analysis

Proteins from synovial tissues of the ankle joint were extracted with RIPA lysate (DingGuo, Beijing, China) and subjected to western blot analysis for protein concentration measurements. Proteins were separated by 10–12% sodium dodecyl sulfate, sodium dodecyl sulfate-polyacrylamide gel electrophoresis and were electroblotted onto a polyvinylidene fluoride membrane and blocked with 5% Skimmed milk powder in Tris-buffered saline containing Tween 20 and subsequently incubated overnight with the appropriate primary antibody at 4°C as follows: NLRP3, Caspase-1, ASC, IL-1β, NF-κB p65 (1:1000, ABclonal, Wuhan, China), and β-actin(1:100,000, ABclonal, Wuhan, China). Membranes were incubated with a horseradish peroxidase-conjugated secondary antibody (1:3000, ABclonal, Wuhan, China) for 1 h. Protein bands were visualized with an enhanced chemiluminescence detection system (Tianneng, Shanghai, China). Then, the ImageJ software was used to semi-quantize the intensity of the strip. After the developed image is converted into a grayscale image, the influence of the image’s background was eliminated and the quantitative parameters and unit (pixel) were set. The WB picture was converted into bright bands; after measuring all the bands and the target protein’s quantitative result = the gray value of the target protein/the gray value of the internal reference protein, the data was sorted and analyzed statistically.

### Immunohistochemistry Analysis

Sections were mounted on slides and fixed in acetone for 10 min, washed in PBS for 5 min, and then incubated with 3% catalase for 25 min to inhibit endogenous peroxidase. The slides were sequentially incubated with polyclonal antibodies against NLRP3, Caspase-1, TLR4, NF-κB p65, and MyD88 (1:100, ABclonal, Wuhan, China) overnight at 4°C. After the primary antibody incubation, the slides were incubated with appropriate second antibodies (1:500, ABclonal, Wuhan, China) for 50 min at 37°C. Then the sections were washed with PBS and stained with 3,3-diaminobenzidine (DAB) for 10 min. After that, they were stained with hematoxylin, dehydrated with 95% ethanol, and sealed with neutral resin for examination under a microscope. The positive brown cells in the sections were identified and counted under an inverted microscope. 200×IHC images were quantitatively analyzed by using ImageJ software. The picture was converted to a grayscale picture. After calibration, the measurement parameters were set, the threshold was adjusted, all positive signals in the image were selected to obtain the average density value, and the final result was calculated.

### Statistical Analysis

The measured data were represented by the mean value ± standard deviation (mean ± SD); statistical analysis was performed with ANOVA (repetitive measure analysis of variance or one-way analysis of variance) by LSD-t multiple comparison test (*p* < 0.05). SPSS 18.0 was employed for statistical analysis of all data, and the difference was statistically significant (*p* < 0.05).

## Results

### Effects of BCP on the MSU‐Induced Gouty Arthritis Model

**FIGURE 1:**
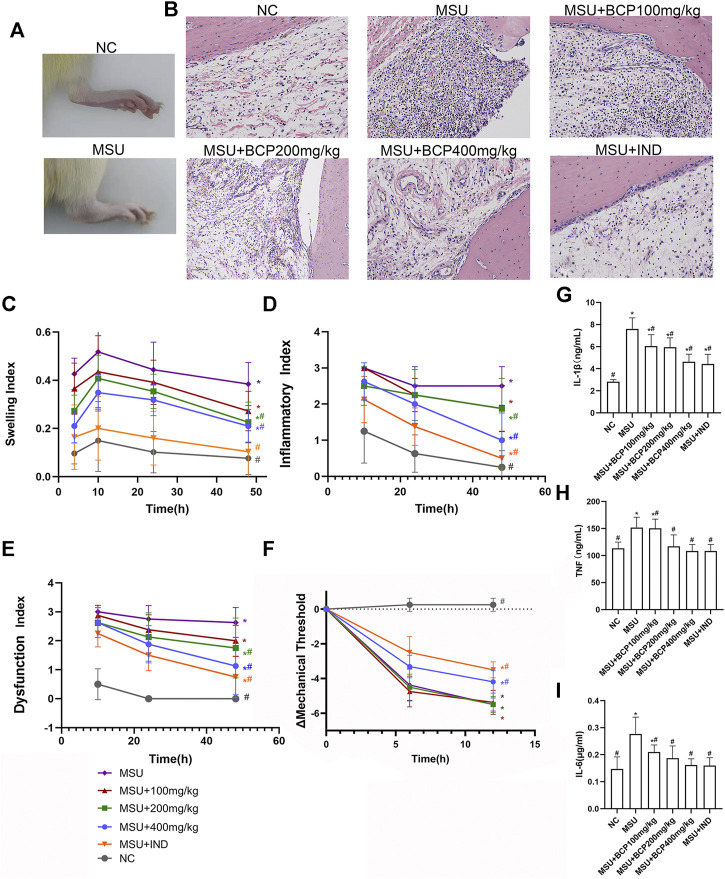
Effects of BCP on the MSU‐induced gouty arthritis model **(A)** Representative photograph of the rat ankles (normal control group and model group) at 48 h following MSU injection **(B)** H and E sections of the ankle synovial tissues after 48 h MSU injection **(C)** Time course of changes in MSU-induced ankle swelling index (*n* = 8, after the Mauchly sphere test, the hypothesis of spherical symmetry was not satisfied (*p* < 0.05); the H-F method was selected to test the difference between groups; the multiple comparisons of repeated measures analysis of variance used the LSD-t method.) **(D)** Time course of changes in MSU-induced ankle inflammatory index (*n* = 8, after the Mauchly sphere test, the hypothesis of spherical symmetry was not satisfied (*p* < 0.05); the H-F method was selected to test the difference between groups; the multiple comparisons of repeated measures analysis of variance used the LSD-t method.) **(E)** Time course of changes in MSU-induced ankle dysfunction index (*n* = 8, in line with the Mauchly sphere test, the multiple comparisons of repeated measures analysis of variance adopted the LSD-t method.) **(F)** Time course of changes in MSU-induced threshold (*n* = 8, after the Mauchly sphere test, the hypothesis of spherical symmetry was not satisfied (*p* < 0.05); the H-F method was selected to test the difference between groups; the multiple comparisons of repeated measures analysis of variance used the LSD-t method. **(G)** The production of IL-1β in rat serum (*n* = 8, one-way analysis of variance is used, and the LSD-t method is used for pairwise comparison) **(H)** The production of TNF in rat serum (*n* = 8, one-way analysis of variance was used, and the LSD-t method was used for pairwise comparison) **(I)** The production of IL-6 in rat serum (*n* = 8, ne-way analysis of variance was used, and the LSD-t method was used for pairwise comparison) *p<0.05 vs. NC group; #*p*<0.05 vs. MSU group. Liver histology and serum markers of liver damage.

During the study, the effect of β-Caryophyllene on ankle swelling and acute gouty arthritis anti-inflammatory effects *in vivo* was studied. A rat model of acute gouty arthritis was established by injecting MSU crystals into the articular cavity of the right ankle. Compared with normal rats, MSU-induced ones significantly swelled around the ankle joint, and the degree of swelling had spread to the limbs outside the joint. (Figure 1A); Compared with the normal control group, it is demonstrated in histological analysis that lymphocytes infiltration in joint synovial tissue (Figure 1B) could be found in the gout group. Based on the analysis, the intervention of β-caryophyllene and indomethacin reduced the recruitment of MSU-stimulated inflammatory cells to the synovial tissue (Figure 1B). Concerning the ankle swelling index of the control group and the treatment group, it was calculated through measuring the ankle circumference. Moreover, the ankle inflammation index and dysfunction index were evaluated with Coderre scale. The degree of swelling of joint swelling was last for 4–48 h and reached a peak 10 h after MSU induction (Figure 1C). The degree of joint swelling, inflammatory index, and dysfunction index gradually decreased over time after 10 h (Figures 1D,E). Compared with the model group, the β-caryophyllene high-dose and middle-dose groups (200 and 400 mg/kg) and indomethacin control group had alleviated the degree of swelling, inflammation index, and dysfunction index evidently (*p* < 0.05). No obvious difference showed up between the β-caryophyllene low dose group (100 mg/kg) and the model group (*p* > 0.05). The difference in mechanical threshold was used to measure the pain sensation of the ankle joint in rats. Compared with the model group, the middle and low doses of β-caryophyllene (200, 100 mg/kg) had no obvious analgesic effect (Figure 1F). However, the high-dose group (400 mg/kg) had obvious analgesic effect. Moreover, the indomethacin control group had good analgesic effect (*p* < 0.05). The analgesic effect of high-dose β-caryophyllene was weaker than that of indomethacin.

After being intervened by β-caryophyllene, the inflammation index and dysfunction index changed with the time (*p* < 0.05). Moreover, blood was collected and subjected to ELISA. According to the results, β-caryophyllene dose-dependently reduced the MSU-induced release of IL-1β, IL-6, as well as TNF from 200 to 400 mg/kg (Figures 1G–I).

**FIGURE 2:**
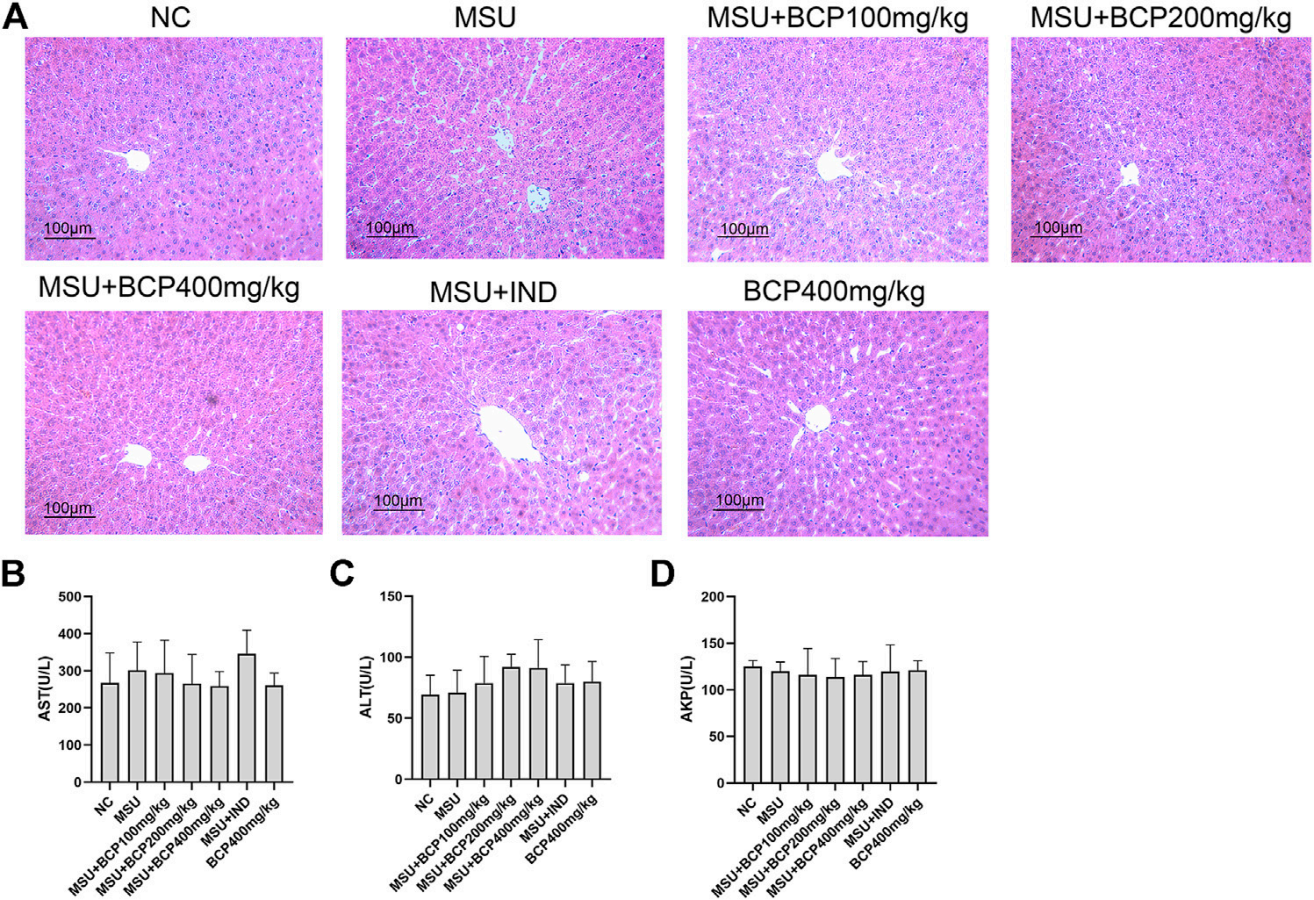
Liver histology and serum markers of liver damage **(A)** H and E sections of the liver tissues 48 h after MSU injection (200X) **(B)** The production of AST in rat serum (*n* = 8, one-way analysis of variance) **(C)** The production of ALT in rat serum (*n* = 8, one-way analysis of variance) **(D)** The production of AKP in rat serum (*n* = 8, one-way analysis of variance). NLRP3 inflammasome and NF‐κB signaling are vital mechanisms of GA.

The serum AST, ALT, and ALP activities were measured to assess whether the treatment with β-caryophyllene could result in liver damage. After MSU treatment, no significant change occurred concerning AST, ALT, and ALP levels. Furthermore, treatment with β-caryophyllene or indomethacin in arthritic rats did not affect these parameters, and normal rats treated with caryophyllene (400 mg/kg) also did not show obvious abnormalities (*p* > 0.05) (Figure 2A). Based on the histological analysis, it failed to reflect that significant changes happened in liver morphology before and after MSU modeling, nor did β-caryophyllene and indomethacin damage gout rats’ liver (Figures 2B–D).

**FIGURE 3:**
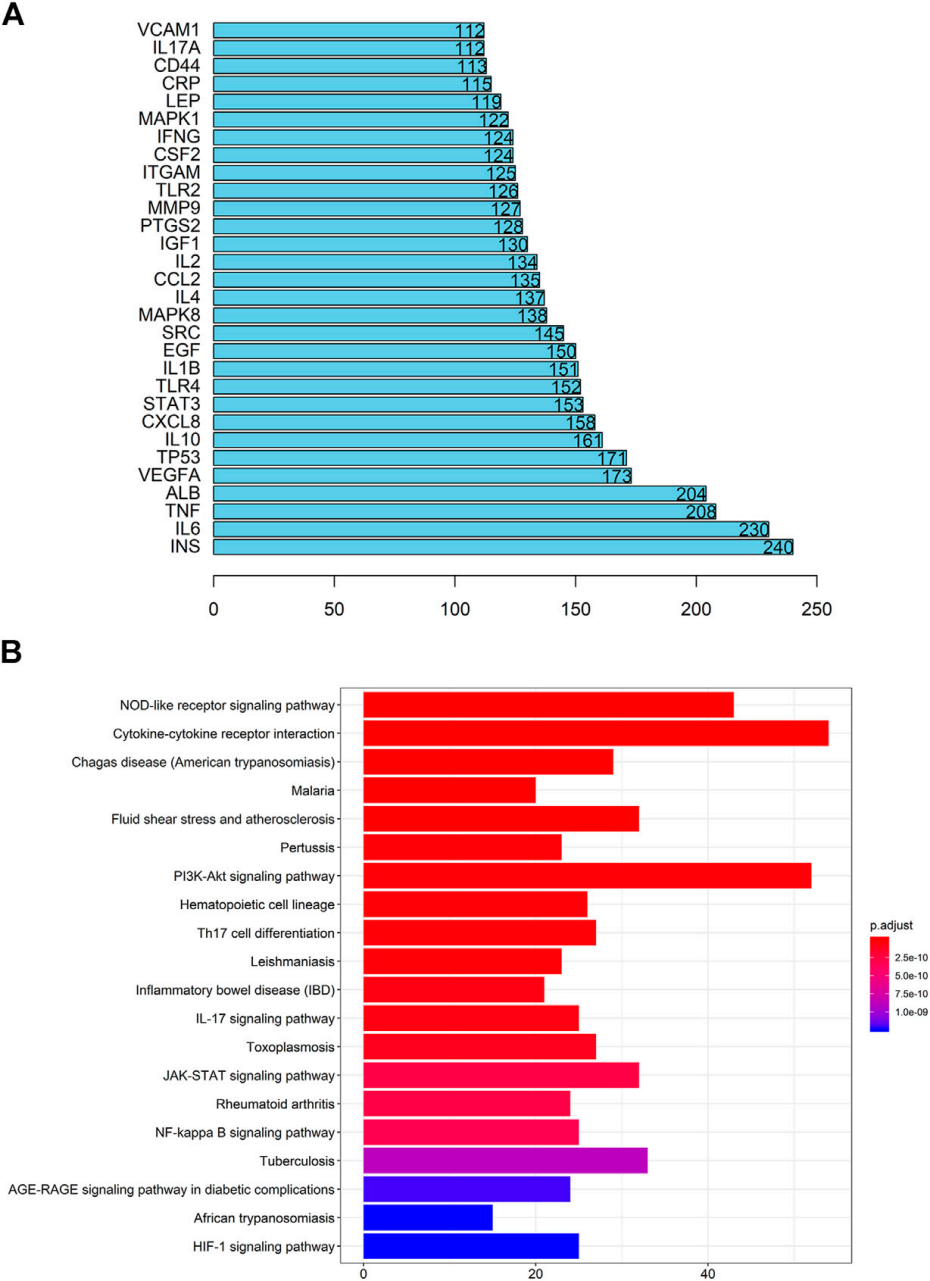
Go analysis, KEGG analysis, and PPI network of gout-inflammation related targets **(A)** Key proteins of the PPI network of gout related targets **(B)** KEGG analysis of gout-inflammation related targets.

**FIGURE 4:**
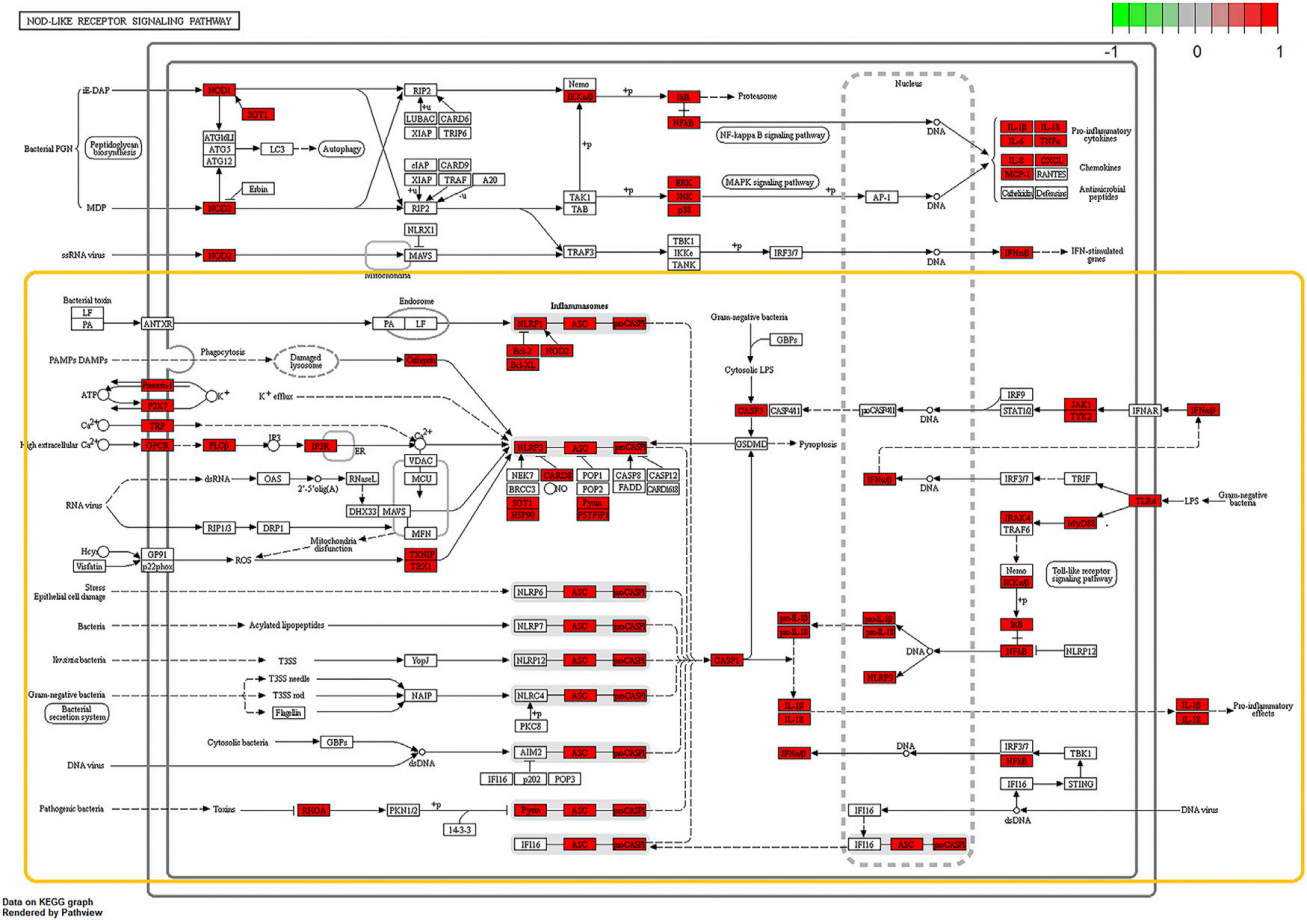
Schematic diagrams of core pathways by KEGG enrichment analysis, NOD-Like Receptor signaling pathway and NF-κB signaling pathway and notably expressed genes (Red rectangles represent significantly expressed genes; The yellow box circles the complete signal path). Prediction of BCP binding to NLRP3 and NF‐κB signaling.

According to the OMIM database and the Genebank database, a total of 825 gout-related genes and 9,006 inflammation-related genes were retrieved. And 534 target genes were obtained bycross-screening. A PPI network related to gout and inflammation was constructed based on the STRING database (Supplementary Datasheet S3). Meanwhile, the top 30 key proteins were screened. Among them, the top five key proteins included INS, IL-6, TNF, ALB, and VEGFA (Figure 3A). It is reflected in the PPI network that cell cycle changes, collagen breakdown, remodeling, and angiogenesis are also related to the occurrence of gout in addition to inflammatory mediator’s high expression. It is confirmed in GO analysis that the former five are highly related to the inflammatory process of gout. Moreover, they focus primarily on cytokine-receptor interactions and angiogenesis (Supplementary Datasheet S4), including receptor-ligand activity, cytokine receptor binding, cytokine activity, growth factor receptor binding, and protein tyrosine kinase activity. To add up, KEGG analysis and GO analysis also proved that the gout-caused process was closely related to the inflammatory response. According to results of the first five analysis: NOD-like receptor signaling pathway, cytokine-cytokine receptor interaction, trypanosomiasis (American trypanosomiasis) Shear stress and atherosclerosis in malaria turned out to be convincing (Figure 3B). Besides, the results indicated that the NF-κB signaling pathway and the Toll-like receptor signaling pathway were essential in the mechanism of the disease (Figure 4, Supplementary Datasheet S5).

**FIGURE 5:**
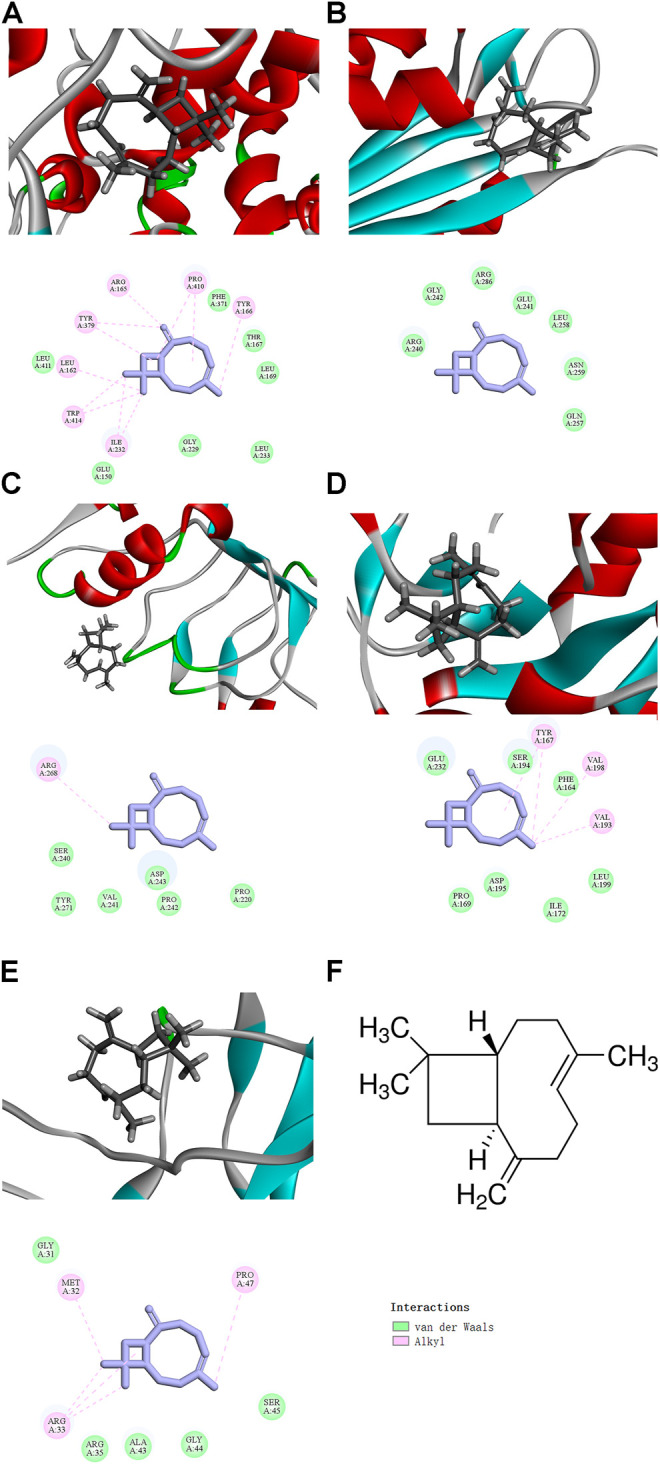
The docking results of BCP binding to NLRP3 and NF‐κB signaling pathway-related targets (The 3D interaction plot and 2D interaction plot of NLRP3 **(A)**, Caspase1 **(B)**, TLR4 **(C)**, MyD88 **(D)**, NF-κB p65 **(E)**; The upper part is a 3D interaction plot, and the lower part is a 2D interaction plot **(F)** The molecular Graph of β-caryophyllene. Inhibition of NLRP3 and NF‐κB signaling expression by BCP.

We used the CDOCKER module in silico. The docking results showed that β-caryophyllene bound to the active sites of NLRP3, Caspase-1, TLR4, Myd88, and p65, with a significant “-CDOCKER energy” (Table 1), and the docking results of β-caryophyllene and these proteins were obtained. In the 3D interaction plot, the gray bar graph is β-caryophyllene, and the binding amino acids in the active pocket are marked in the 2D interaction plot (Figures 5A–F). β-Caryophyllene has alkylation with Arg165, Pro410, Tyr166, Ile232, Trp414, leu162, ty379 and has van der Waals force effects with Phe371, Thr167, Leu169, Leu233, Gly229, Glu150, and Leu411 of NLRP3; has van der Waals force effects with Arg240, Gly242, Arg286, Glu241, Leu258, Asn259 and Gln257 of Caspase1; has alkylation with Arg268, and has van der Waals force effects with Ser240, Tyr271, Val241, Asp243, Pro242, Pro220 of TLR4; has alkylation with Tyr167, Val198, Val193, and has van der Waals force effects with Glu232, Ser194, Phe164, Leu199, Ile172, Asp195, and Pro169 of MyD88; has alkylation with Met32, Pro47, Arg33, and van der Waals force effects with Gly31, Arg35, Ala43, Gly44, Ser45 of p65 (Figures 5A–F). These data proved that β-caryophyllene has a good binding effect with NLRP3 inflammasome components and NF-κB signaling pathway-related proteins, which may be targeted for β-caryophyllene to exert anti-inflammatory effects in gout.

**TABLE 1:** CODOCKER Interaction Energy of β-caryophyllene on targets of gouty arthritis.

Gene	PDB ID	-CODOCKER interaction energy (°kcal/°mol)
NLRP3	6NPY	31.92
TLR4	2Z62	18.71
MyD88	4DOM	19.96
Caspase-1	2H4W	16.00
NF-κb p65	1VKX	15.74

**FIGURE 6:**
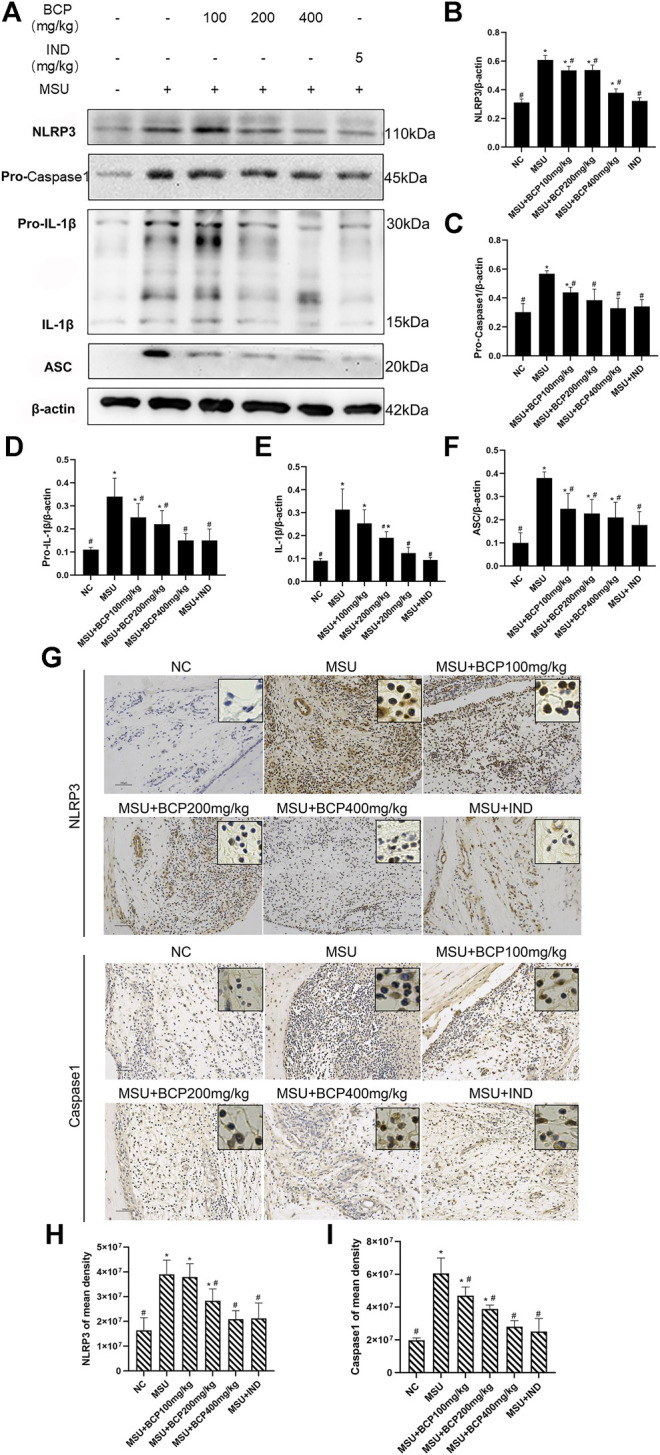
Inhibition of NLRP3 signaling expression in synovial tissues of gout rat ankles by BCP. **(A)** The results of NLRP3, Pro-Caspase1, IL-1β (Pro-IL-1β, IL-1β), and ASC by western blot **(B)** Protein expression levels of NLRP3 by WB **(C)** Protein expression levels of Pro-Caspase1 by WB. **(D)** Protein expression levels of Pro-IL-1β by WB **(E)** Protein expression levels of IL-1β by WB **(F)** Protein expression levels of ASC by WB **(G)** NLRP3 IHC stains (200×and 400×) **(H)** Protein expression levels of NLRP3 by IHC **(I)** Caspase1 IHC stains (200×and 400×) **(I)** Protein expression levels of Caspase1 by IHC *p<0.05 vs. NC group; #*p*<0.05 vs. MSU group, One-way analysis of variance is used, and the LSD-t method is used for pairwise comparison.

To analyze the effect of β-caryophyllene on MSU-induced inflammatory response *in vivo*, a rat model of acute gouty arthritis was established. Interestingly, it was found that β-caryophyllene inhibited IL-1β and Pro-IL-1β secretion in rat synovial tissue in a dose-dependent manner through western blot and the expression levels were significantly suppressed in the 100, 200, 400 mg/kg MSU + BCP groups compared to that in the MSU group (all, *p* < 0.05) (Figures 6A,D,E). IL-1β is critical in the inflammatory response, and its maturation is associated with the NLRP3 inflammasome. In this case, effects of β-caryophyllene on the expression of the NLRP3 inflammasome relative components (NLRP3, Caspase1, and ASC) in synovial tissue were investigated. And the expression levels of NLRP3 inflammasome relative components (NLRP3, Pro-Caspase1, and ASC) were significantly suppressed in the 100, 200, 400 mg/kg MSU + BCP groups compared to that in the MSU group (all, *p* < 0.05) (Figures 6A–F). Consequently, it was drawn that β-caryophyllene reduced the secretion of NLRP3, Caspase-1, and ASC in the synovial tissue of MSU-induced rats. The protein expression of NLRP3 and Caspase-1 in synovial tissues was measured as well by immunohistochemistry. And β-caryophyllene was proved to inhibit the nuclear expression of these proteins (Figures 6G–I).

**FIGURE 7:**
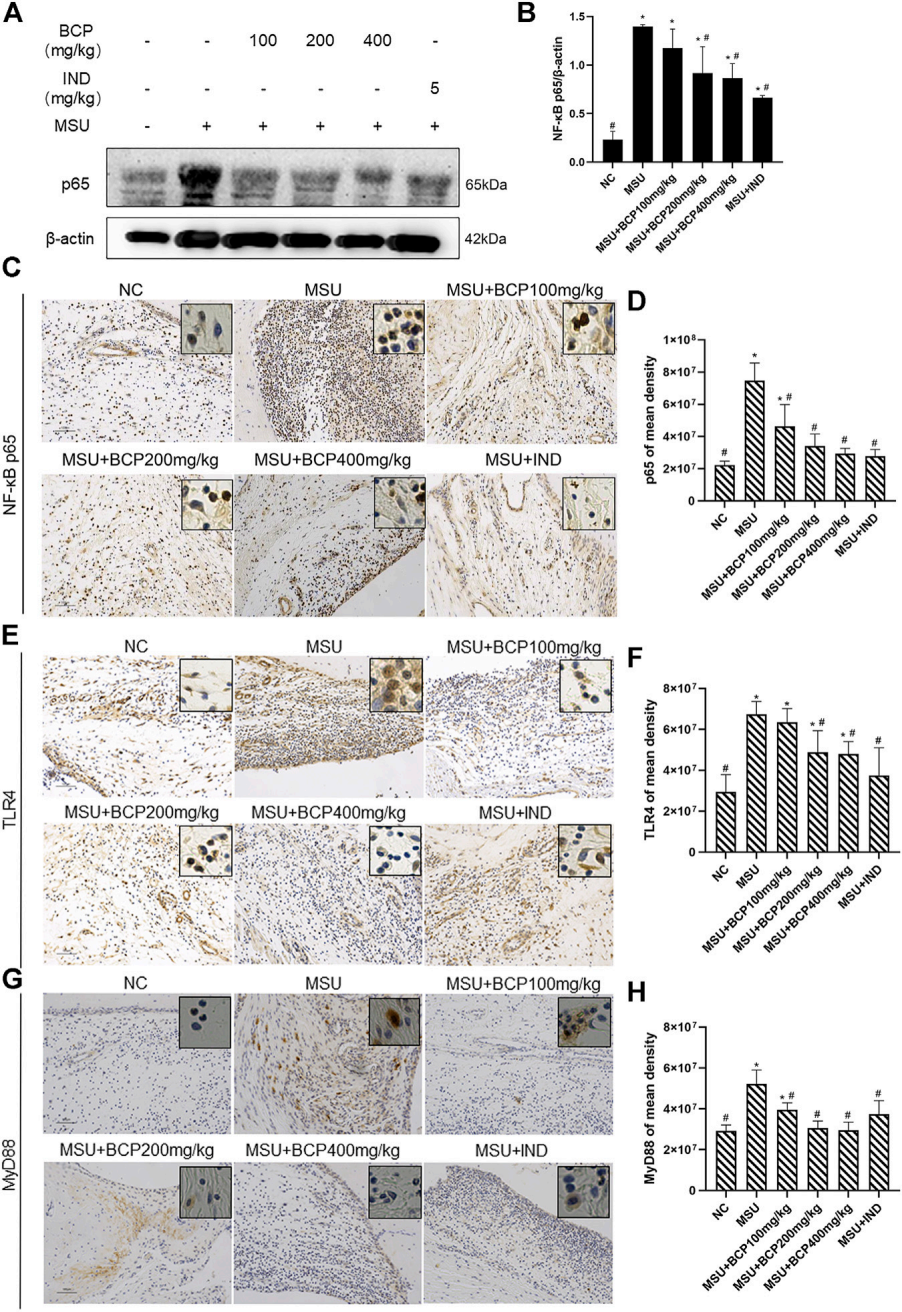
Inhibition of NF-κB signaling expression in synovial tissues of gout rat ankles by BCP **(A)** The results of NF-κB p65 by western blot **(B)** Protein expression levels of NF-κB p65 by WB **(C)** NF-κB p65 IHC stains (200×and 400×) **(D)** Protein expression levels of NF-κB p65 by IHC **(E)** TLR4 IHC stains (200×and 400×) **(F)** Protein expression levels of TLR4 by IHC **(G)** MyD88 IHC stains (200×and 400×) **(H)** Protein expression levels of MyD88 by IHC *p<0.05 vs. NC group; #*p*<0.05 vs. MSU group. One-way analysis of variance is used, and the LSD-t method is used for pairwise comparison.

Based on the KEGG analysis (Figures 4A,B) carried out previously, it was found that NLRP3-related molecular signal transduction had connections with TLR4/NF-κB signal transduction, and NF-κB-related protein expressions were conducive to IL-1β′s maturation. Moreover, we conjectured that β-caryophyllene exerted an effect on TLR4/NF-κB signal transduction. Later, through immunohistochemistry analysis and Western blot, these proteins were detected. The results proved that β-caryophyllene dose-dependently inhibited the up-regulation of TLR4/NF-κB signaling pathway-related proteins (TLR4, MyD88, and p65) in synovial tissue after MSU-stimulation and the expression levels of p65 were significantly suppressed in the 200,400 mg/kg MSU + BCP groups compared to that in the MSU group (all, *p* < 0.05) (Figures 7A–H). What’s more, it also managed to reduce nuclear expression. All these results have jointly demonstrated that β-caryophyllene reduced IL-1β secretion, thus alleviating acute gout arthritis by inhibiting NLRP3 inflammasome production and reducing TLR4/NF-κB signaling transduction.

## Discussion

Gout is most common in patients with acute onset symptoms and/or peri-articular swelling and rapid onset (12–24 h) (Abhishek et al., 2017), which is characterized by pain, redness, and distal joint swelling. People with this disease can have a serious impact on their life quality and mental health. Taking this into account, this study investigates the effects of β-caryophyllene on MSU-induced acute arthritis of inflammation. Consistent with other studies, inflammation activation-related pathways are critical to the development of gout. However, their specific signal transduction pathways and key protein targets are not definite (So and Martinon, 2017). Thanks to the rapid development of bioinformatics, these problems have been gradually addressed. When carrying out the study, we conducted GO analysis on the differential expression of genes in gout and inflammation, then found that these genes were strongly focused on cytokine receptor binding, G protein-coupled receptor binding, nuclear receptor activity, and transcription factor activity, and all results conformed to characteristics of transcription factor activation in other studies (Russo, 2011; Ling and Bochu, 2014). Based on KEGG signal pathway enrichment analysis, it was proposed that the interaction between cytokine-cytokine receptors, nod-like receptor signaling pathway, tumor necrosis factor signaling pathway, and NF-κB signaling pathway enrichment is the pillar of previous studies, which is of great significance (So and Martinon, 2017). Although oxidative stress fails to score high in our bioinformatics research, the release of reactive oxygen species (ROS) in synovial cells due to the deposition of MSU crystals drives the inflammatory pathway and oxidative stress response (Abhishek et al., 2017). Synovial cells, monocytes, and neutrophils also induce the production of cytokines through nicotinamide adenine dinucleotide phosphate (NADPH) and Inducible nitric oxide synthase (iNOS). Consequently, it increases the production of reactants (such as superoxide anion and nitric oxide) and indirectly exacerbates local oxidative stress levels resulting in cell apoptosis (Zamudio-Cuevas et al., 2015; Manchope et al., 2016; Ruiz-Miyazawa et al., 2017).

Although drugs are currently available for the treatment of gouty arthritis, there have been reports of adverse reactions (Finkelstein et al., 2010). In particular, hepatotoxicity is an important issue associated with drugs and dietary supplements. As for natural compounds, they embrace lots of health-promoting effects, such as anti-inflammatory, antioxidant, and immune-regulating effects (Rahman et al., 2006). It is noted in previous studies that natural compounds and botanical herbal extracts treat inflammation due to MSU crystals (Russo, 2011; Ling and Bochu, 2014). β-caryophyllene is taken as a natural sesquiterpene with the aforementioned outstanding biological activity. Based on our interactions with β-caryophyllene, a natural product, and several key target proteins in the PPI network, and combined with KEGG enrichment analysis results, it was discovered that β-caryophyllene is highly bound to the Toll-like receptor pathway and the NF-κB pathway.

The model of acute gouty arthritis was mainly established in rats and mice. Moreover, the stimulation of the synovial membrane and surrounding tissues by MSU crystals was realized by injecting sodium urate solution into the ankle joint cavity (Zhou et al., 2018). However, in some studies, they used mouse hind limb knee joint as the common test objection (Vieira et al., 2015). When carrying out this study, we injected MSU suspension into the rat’s ankle joint cavity. With the aid of ankle joint, it manages to effectively simulate the attack of acute gouty arthritis in humans. In general, it is easier and more likely to be successful to operate on rats than mice. In the acute phase of gouty arthritis, MSU precipitated in synovial fluid when neutrophils and macrophages accumulated and infiltrated on the synovium (Desai et al., 2017). Despite of this, after the acute attack period, especially after drugs were used, the acute inflammation level would decrease within a short period of time and transform to chronic inflammation. Under that circumstance, more lymphocyte infiltration showed up, part of the synovial epithelium lost, and fibrous tissue became hyperplasia (Li et al., 2019). The rodents (rats in this study) contain uricase which does not belong to human beings, and the degradation metabolism of MSU is faster. And this time, the acute inflammation gradually subsides, so the lymphocyte infiltration would become more obvious in our results. When conducting our study, the protocol of pre-administration prior to modeling was chosen. Later, an administration happened after modeling, thus reflecting the efficacy of BCP on acute changes of gouty arthritis, which is also a common choice for most similar studies (Wu et al., 2018; Li et al., 2019).

Alanine aminotransferase (ALT), aspartate aminotransferase (AST), and alkaline phosphatase (ALP) serve as significant indicators in detecting liver cell damage. So long as liver cells get damaged and the liver function becomes abnormal, these indicators in serum will increase. Based on a report in a former study (Li et al., 2019), we explored a relatively conservative dose out of safety. And Indomethacin is deemed as a common drug for gout and acute arthritis. The conservative β-caryophyllene dose may be less effective than indomethacin. In our study, β-caryophyllene failed to increase the levels of ALT, AKP, and ALP in serum, implying that β-caryophyllene did not result in changes in liver function when carrying out the experiment. In a study of sub-chronic toxicity, β-caryophyllene (700 mg/kg/ d) was administered orally to rats for 90 days without any significant toxicological manifestations (Schmitt et al., 2016). Subsequent studies sought to study effects of doses of 800 mg and above when testing its toxicity. In our opinion, it is possible that the bioavailability of β-caryophyllene exerts a positive/negative impact on drugs action. Therefore, in the coming future, the anti-inflammatory effect on gouty arthritis will be further studied so as to explore the effect of β-caryophyllene complexes (at a dose of 200 or 400 mg/kg, once a day). Specifically, they are complexes with cyclodextrins, liposomes, and nanoparticles which work to improve bioavailability (Santos et al., 2018).

A growing number of molecular docking experiments have been applied in the study of natural compounds. Quercetin specifically interacted with different residues of COX-1 and COX-2 through molecular docking experiments. Therefore, the inhibitory effect of quercetin on cyclooxygenase *in vivo* experiments mentioned in previous studies got verified (Lescano et al., 2018). Phytochemicals (such as curcumin and rutin) inhibited α-glucosidase and α-amylase managed to be screened through molecular docking. Furthermore, they were verified by experiments *in vivo* and *in vitro*. Obviously, these compounds effectively reduced blood sugar by inhibiting α-glucosidase and α-amylase (Riyaphan et al., 2018). In this study, based on the molecular docking experiments, it was found that β-caryophyllene embraced interaction energy with NLRP3, Caspase-1, ASC, TLR4, MyD88, and NF-κB p65, among which NLRP3 is supposed to embrace the highest interaction energy. All these results have jointly implied that β-caryophyllene is most likely be associated with NLRP3, and it is more possible to be NLRP3 inflammasomes components (NLRP3, Caspase1, ASC) and NF-κB signaling pathway (TLR4, MyD88, NF-κB p65). Moreover, β-caryophyllene may act on the NLRP3 inflammasome and NF-κB pathway with a multi-target effect, thus providing guidance for subsequent animal experiments.

Furthermore, based on our results, it is indicated that β-caryophyllene effectively reduced the inflammatory factor IL-1β in rat models of inflammatory arthritis, inhibited the assembly and activation of NLRP3 inflammasomes, as well as reduced the activities of Toll-like receptors and NF-κB pathways in gout rats, which conform to the previous studies (Chen et al., 2019; Irrera et al., 2019). In an open proof-of-concept phase 2a clinical trial for male gout patients, dapansutrile was first discovered to be a specific NLRP3 inflammasome inhibitor with satisfactory safety and efficacy in reducing joint pain (Klück et al., 2020). β-Caryophyllene decreased the productions of TNF and IL-6 in the serum of gout rats, thus relieving inflammation symptoms. And these two inflammatory factors were found in our gout-inflammation PPI network as well. The multi-target effect of β-Caryophyllene inhibited the activation of two gout-inflammation pathways screened in our KEGG analysis---the NLRP3 inflammasome and the NF-κB pathway. And in molecular docking experiments, it was observed that BCP is significantly related to NLRP3, Caspase1, TLR4, MyD88, and NF-κBp65. All these have been verified by Western blot and immunohistochemistry. It must be pointed out that the mechanism of action of BCP on gouty arthritis is the same as the current clinical diagnosis and treatment measures, that is, to reduce the inflammatory response and reduce the symptoms of gouty arthritis (redness, swelling, heat, pain) by inhibiting the inflammatory response. As for the cause of gouty arthritis-the disorder of uric acid metabolism, the positive effect of BCP has not been observed, which may be related to the mode BCP acts. So far, no studies have been conducted on the positive effect of BCP on the pathways and enzymes related to uric acid metabolism. However, when gouty arthritis attacks, it is often accompanied by the pain. β-caryophyllene is a phytocannabinoid that exerts analgesic effects by activating cannabinoid type 2 receptors (CB2), possibly through the mechanism that activates CB2 receptors and decreases the level of phosphorylated extracellular regulated protein kinase 1/2 (ERK1/2), so as to inhibit 2′-3′-dideoxycytidine (ddC, zalcitabine)-induced up-regulation of proinflammatory cytokine expression and mechanical allodynia (Aly et al., 2019). During the onset of gouty arthritis, pain symptoms are so obvious that it is reasonable to speculate that β-caryophyllene may also have analgesic effects on gouty arthritis. Moreover, based on some studies, the activation of NLRP3 inflammasome can be inhibited by the CB2 receptor, thus relieving the inflammatory pain (Gao et al., 2018). Theoretically, β-caryophyllene is likely to inhibit NLPR3 inflammasome by inhibiting the NLRP3 receptor directly and activating the CB2 receptor indirectly on anti-inflammatory and analgesic effect. Further researches on β-caryophyllene also proceed on the CB2 receptor and the effect on NLRP3. Furthermore, we will seek to illustrate the synergistic treatment of β-caryophyllene through analgesic and anti-inflammatory effects, thus relieving gouty arthritis symptoms. As for limitations of our study, we failed to explore the specific mechanism of β-caryophyllene at the level of RNA transcription. What’s more, rat models were established merely by MSU crystals. Therefore, we need to further investigate these issues in the future.

## Data Availability

The datasets presented in this study can be found in online repositories. The names of the repository/repositories and accession number(s) can be found in the article/Supplementary Material.
