# Supplementary figures and images for: β-Caryophyllene Ameliorates MSU-Induced Gouty Arthritis and Inflammation Through Inhibiting NLRP3 and NF-κB Signal Pathway: In Silico and In Vivo
Source: Front Pharmacol. 2021 Apr 23;12:651305. doi: 10.3389/fphar.2021.651305 (PMC8103215; doi:10.3389/fphar.2021.651305)

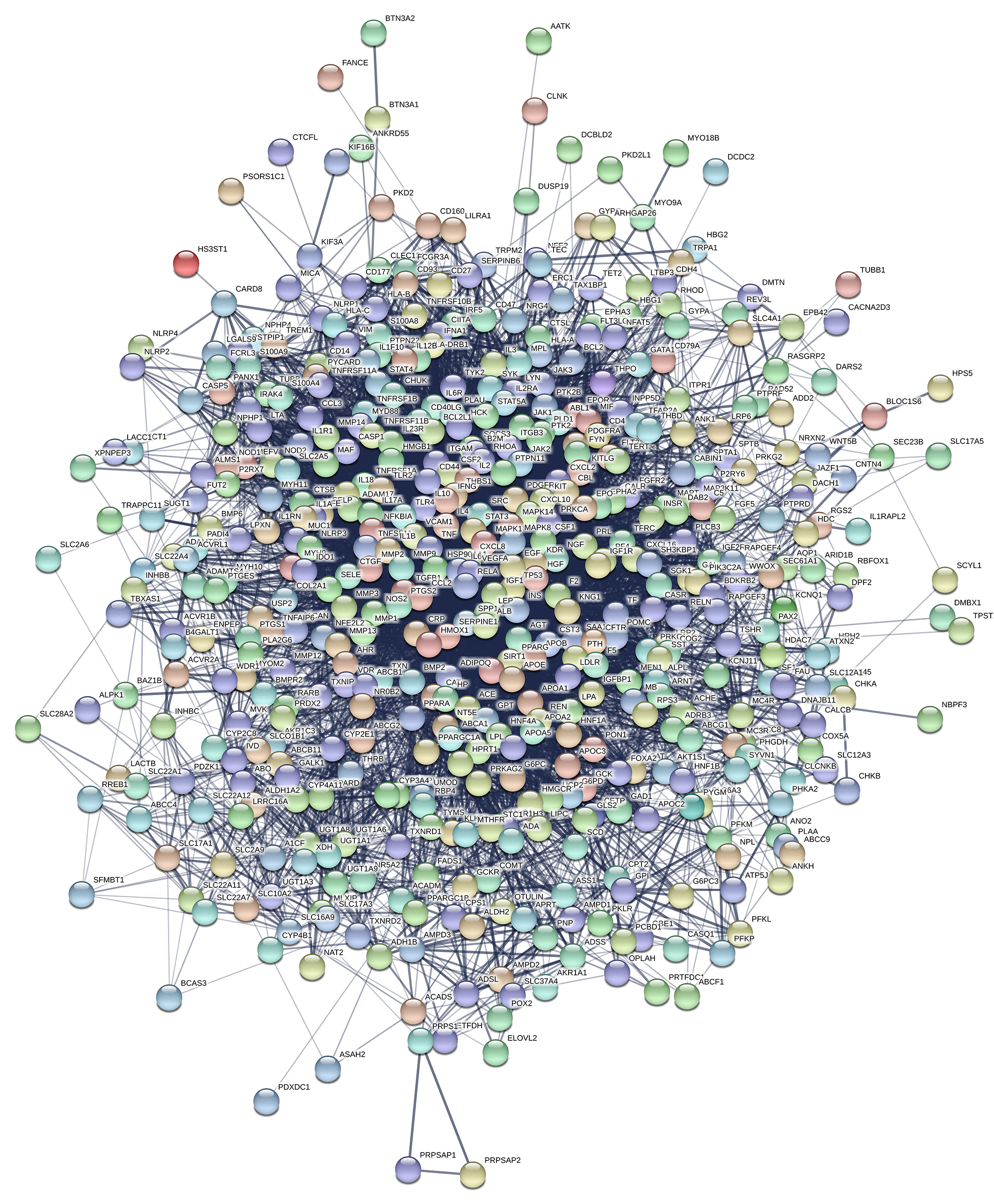

Supplement: Supplementary file 3 [file Image1.TIF]

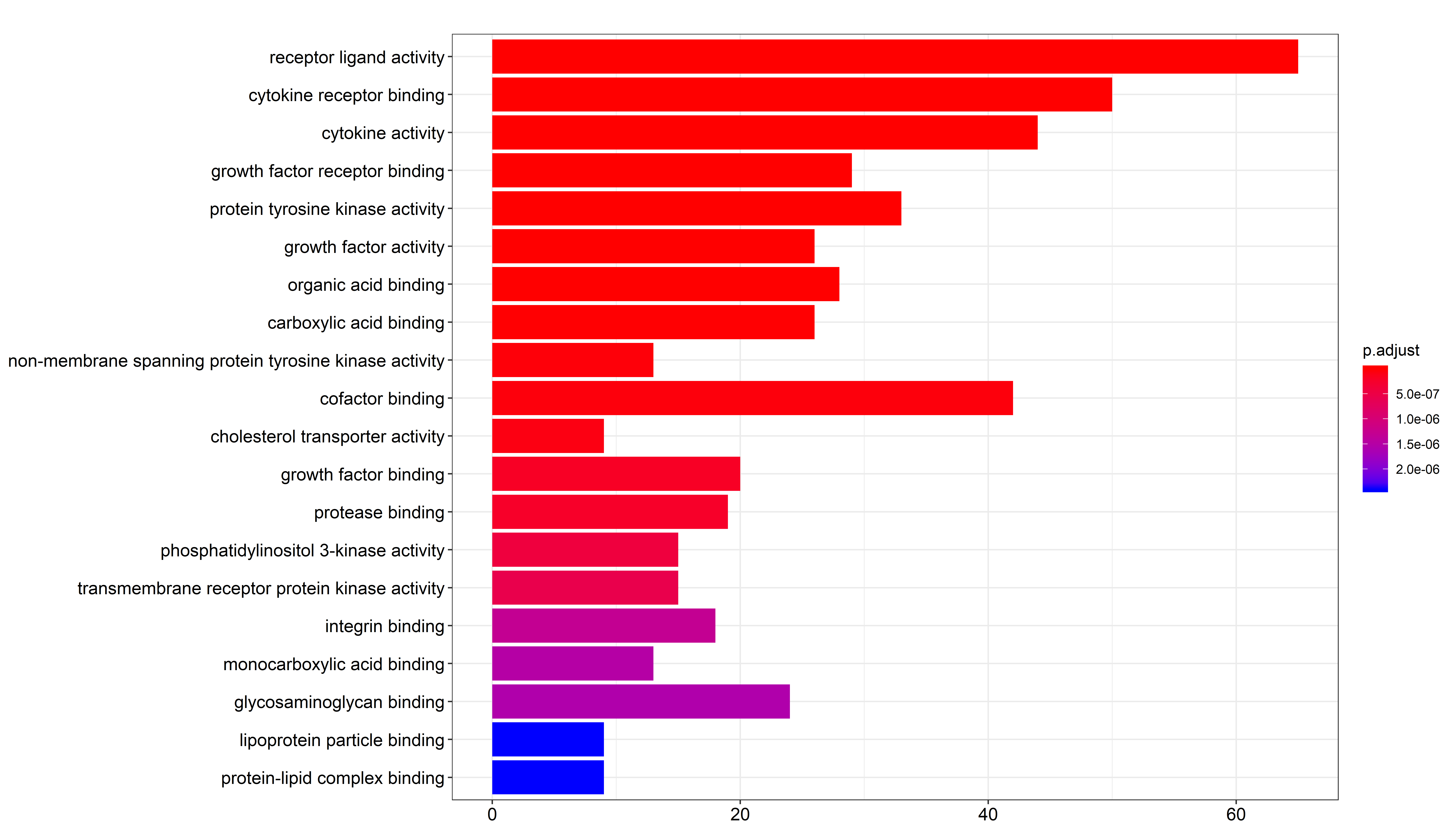

Supplement: Supplementary file 5 [file Image2.TIFF]
