# Supplementary material for: β-Caryophyllene Ameliorates MSU-Induced Gouty Arthritis and Inflammation Through Inhibiting NLRP3 and NF-κB Signal Pathway: In Silico and In Vivo
Source: Front Pharmacol. 2021 Apr 23;12:651305. doi: 10.3389/fphar.2021.651305 (PMC8103215; doi:10.3389/fphar.2021.651305)
Supplement: Supplementary file 4 [file DataSheet1.PDF]

Industriestrasse 25, CH-9471 Buchs (SG), Switzerland
Tel: +41 81 755 2511 Fax: +41 81 756 5449

Certificate of Analysis

**Product Name:**	(–)-*TRANS*-CARYOPHYLLENE<br>puriss.
**Product Number:**	22075
**Product Brand:**	Fluka
**Molecular Formula:**	C15H24
**Molecular Mass:**	204.35
**CAS Number:**	87-44-5

TEST	SPECIFICATION	LOT BG885334V RESULTS
**APPEARANCE (COLOR)**	COLORLESS	COLORLESS
**APPEARANCE (FORM)**	CLEAR LIQUID	CLEAR LIQUID
**PURITY (GC AREA %)**	≥ 98.5 % REL	99.5 % REL
**SPECIFIC ROTATION (20/D)**	-10.0 ± 1.0 DEGREES	-10.2 DEGREES
**CONCENTRATION**	--	NEAT
**REFRACTIVE INDEX N20/D**	1.498 - 1.500	1.499
**SOLUBILITY (COLOR)**	--	COLORLESS
**SOLUBILITY (TURBIDITY)**	--	CLEAR (<3.5 NTU)
**SOLUBILITY (METHOD)**	--	1ML IN 10ML ETOH
**INFRARED SPECTRUM**	CORRESPONDS TO STANDARD SPECTRUM	CORRESPONDS

QC RELEASE DATE 08/JAN/18

Edeltraud Schwärzler, Manager
Quality Control
Buchs, Switzerland

Sigma-Aldrich guarantees the 'Sales-Specification' values only, additional lot specific tests may be included for further information. The current 'Sales-Specifications' sheet is available on request. For further inquiries, please contact our Technical Service.Sigma-Aldrich warrants, that its products conform to the information contained in this and other Sigma-Aldrich publications. Purchaser must determine the suitability of the product for its particular use. See reverse side of invoice for additional terms and conditions of sale. The values given on the 'Certificate of Analysis' are the results determined at the time of analysis.
